# DSEception: a noval neural networks architecture for enhancing pneumonia and tuberculosis diagnosis

**DOI:** 10.3389/fbioe.2024.1454652

**Published:** 2024-09-03

**Authors:** Shengyi Li, Yue Hu, Lexin Yang, Baohua Lv, Xue Kong, Guangliang Qiang

**Affiliations:** ^1^ Internet of Things Engineering, Beijing-Dublin international College, Beijing University of Technology, Beijing, China; ^2^ China Academy of Chinese Medical Sciences, Guang’anmen Hospital, Beijing, China; ^3^ Department of Radiology, Taian City Central Hospital, Qingdao University, Qingdao, Shandong, China; ^4^ Department of Thoracic Surgery, Peking University Third Hospital, Beijing, China

**Keywords:** inception, lung diseases, decision making, deep learning, diagnosis

## Abstract

**Background:**

Pneumonia and tuberculosis are prevalent pulmonary diseases globally, each demanding specific care measures. However, distinguishing between these two conditions imposes challenges due to the high skill requirements for doctors, the impact of imaging positions and respiratory intensity of patients, and the associated high healthcare costs, emphasizing the imperative need for intelligent and efficient diagnostic methods.

**Method:**

This study aims to develop a highly accurate automatic diagnosis and classification method for various lung diseases (Normal, Pneumonia, and Tuberculosis). We propose a hybrid model, which is based on the InceptionV3 architecture, enhanced by introducing Deepwise Separable Convolution after the Inception modules and incorporating the Squeeze-and-Excitation mechanism. This architecture successfully enables the model to extract richer features without significantly increasing the parameter count and computational workload, thereby markedly improving the performance in predicting and classifying lung diseases. To objectively assess the proposed model, external testing and five-fold cross-validation were conducted. Additionally, widely used baseline models in the scholarly community were constructed for comparison.

**Result:**

In the external testing phase, the our model achieved an average accuracy (ACC) of 90.48% and an F1-score (F1) of 91.44%, which is an approximate 4% improvement over the best-performing baseline model, ResNet. In the five-fold cross-validation, our model’s average ACC and F1 reached 88.27% ± 2.76% and 89.29% ± 2.69%, respectively, demonstrating exceptional predictive performance and stability. The results indicate that our model holds promise for deployment in clinical settings to assist in the diagnosis of lung diseases, potentially reducing misdiagnosis rates and patient losses.

**Conclusion:**

Utilizing deep learning for automatic assistance in the diagnosis of pneumonia and tuberculosis holds clinical significance by enhancing diagnostic accuracy, reducing healthcare costs, enabling rapid screening and large-scale detection, and facilitating personalized treatment approaches, thereby contributing to widespread accessibility and improved healthcare services in the future.

## Introduction

Pneumonia and tuberculosis are common pulmonary infections with a significant global incidence. Pneumonia is mainly caused by bacteria such as *Streptococcus* pneumoniae and *Mycoplasma* pneumoniae, and since the beginning of the 21st century, it has been one of the most predominant factors of widespread contagions (Torres et al., 2021). Pulmonary tuberculosis is another lung-affected infectious disease transmitted through the air, with which a quarter of the world’s population is infested (Pallett and Houston, 2021). Both these diseases have a considerable impact on individual health and public hygiene. The severity of pneumonia is comprehensive, and some mild symptoms may be resolved with early care, while severe cases might require hospitalization (Sun et al., 2019). Consequently, Identifying and distinguishing between these conditions requires significant attention and careful consideration in the medical field.

Timely diagnosis in the early stages is beneficial for determining early treatment plans and the proper use of medications, which could alleviate the negative impact of pneumonia or tuberculosis on the patient’s physiological health (Driss et al., 2022). X-ray detection is a commonly used method for the current detection of these lung diseases. This method involves taking a chest X-ray (CXR) of the patient’s chest and conducting a comprehensive evaluation of the patient’s clinical history, vital signs, and laboratory tests, which are ultimately assessed by a doctor (Yan and Tao, 2023). However, diagnosing the patient is not straightforward in this process, and difficulties may be displayed in several aspects. Firstly, the entire diagnostic process requires a high level of involvement from physicians, which means that pursuing diagnostic accuracy necessitates that they are supposed to be familiar with the radiographic features of the related diseases (Nambu et al., 2014). Secondly, relying solely on a single CXR cannot directly determine the patient’s condition. Many external factors, such as the location of the imaging and the intensity of patient exhalation, could affect the images presented in CXRs, making it more challenging for doctors to diagnose lung diseases (Yan and Tao, 2023). Concomitantly, unclear images caused by external factors may lead doctors to misjudge bacterial pneumonia and viral pneumonia, resulting in the treatment process being mistakenly guided (Neuman et al., 2012). Moreover, in low-resource countries, medical facilities and resources may not be able to support this type of diagnosis, which is costly in terms of personnel training and equipment (Rahman et al., 2020). In general, it can be analyzed from these imperfections that the overall process of manually diagnosing lung disease by X-ray might be inefficient. Therefore, a more efficient and intelligent diagnostic method with digital technologies is expected to be developed.

**FIGURE 1:**
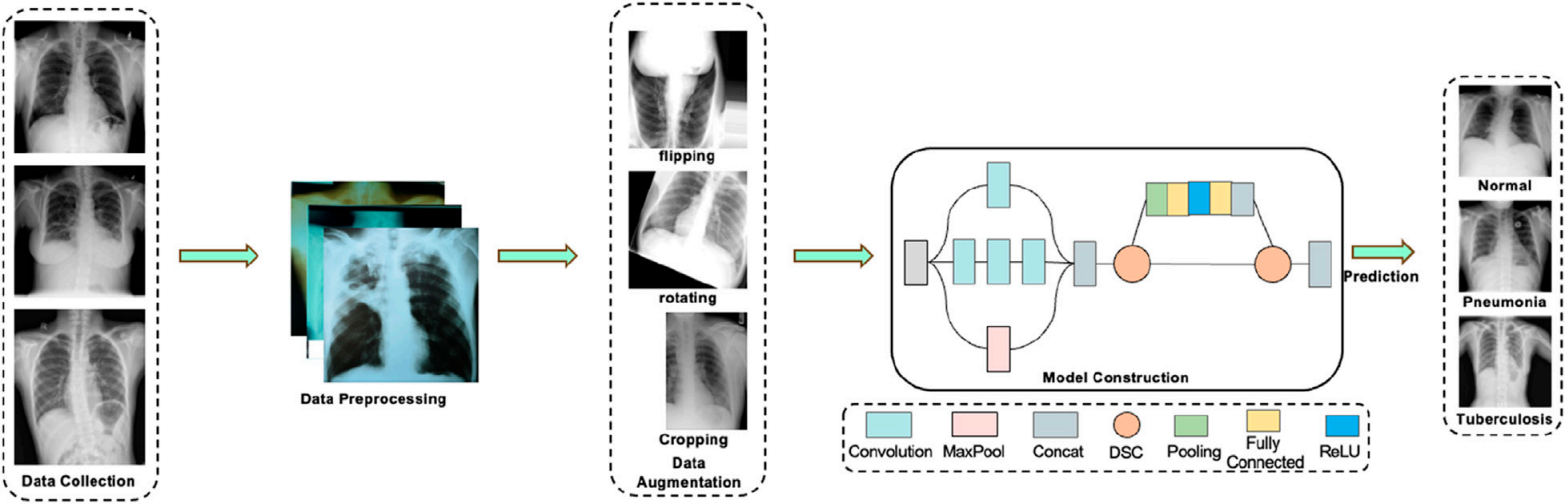
The workflow of this study. The process first involves the collection of data on tuberculosis, pneumonia, and normal lungs. Following that, data preprocessing is carried out, which includes various data augmentation techniques. The preprocessed data is then fed into a deep learning model for prediction, ultimately yielding relatively accurate results for assisted diagnosis.

This study aims to achieve efficient and accurate diagnosis of pneumonia and tuberculosis based on chest X-ray images, proposing the DSEception model. The DSEception model proposed in this paper is based on the Inception architecture, incorporating the Deepwise Separable Convolution (DSC) module and the Squeeze-and-Excitation mechanism from SENet. The DSC module extracts more detailed texture features while reshaping the feature map size without significantly increasing the number of parameters. The Squeeze-and-Excitation mechanism, as a lightweight self-attention mechanism, enhances the model’s representational ability by learning and readjusting the importance distribution of different channels. Compared to the traditional Inception model, DSEception has significant improvements in feature extraction and channel importance learning, further enhancing the model’s diagnostic performance. Additionally, in the initial stages of data preparation, a series of filtering and data augmentation techniques were employed to minimize noise interference and enhance the model’s ability to extract detailed information. These combined efforts effectively improve the model’s predictive accuracy, thereby fulfilling the objective of precisely predicting and classifying different lung diseases. The workflow of this study is illustrated in Figure 1.

## Methods

### Data collection

In the investigation into pulmonary diseases, two significant datasets have been utilized to facilitate the diagnosis and classification of conditions such as pneumonia and tuberculosis (TB) through computational models. The first dataset, curated from various sources, is designed to aid in the classification of pneumonia—an infection leading to the inflammation of one or both lungs, potentially caused by viruses, bacteria, fungi, or other pathogens (https://www.kaggle.com/datasets/tawsifurrahman/tuberculosis-tb-chest-xray-dataset). This dataset is structured to support the development and validation of Convolutional Neural Networks (CNNs), comprising training, testing, and prediction subsets to evaluate the model’s performance. The dataset has been intentionally balanced to simplify model training, although researchers are encouraged to introduce additional data sources and imbalance for a more rigorous analysis, potentially enhancing the CNN model through Data Augmentation techniques. The second dataset, a collaborative effort by researchers from Qatar University, the University of Dhaka, and their partners, focuses on Tuberculosis (TB) detection using chest X-ray images. It juxtaposes 700 publicly accessible TB images and an additional 2800 TB images available through the NIAID TB portal, against 3,500 normal images, thus facilitating a comprehensive analysis of TB-positive cases (https://www.kaggle.com/datasets/vivek468/beginner-chest-xray-image-classification). The dataset sources include the publicly available Montgomery and Shenzhen datasets from the National Library of Medicine (NLM), the Belarus dataset for a drug resistance study, and the NIAID TB portal program dataset, offering a diverse range of TB-positive CXR images for analysis.

### Data preprocessing

To enhance feature visibility and improve the predictive performance of the model, a series of preprocessing operations were applied to all images. Initially, a mean filter was utilized to remove noise from the images. Subsequently, a Gaussian high-pass filter was employed to extract edge information, which was then combined with the original images to make the edges more pronounced. Finally, histogram equalization in the contrast space was conducted, enhancing the representation of detail information and contributing to the improvement of model performance.

Given the presence of data imbalance in the original dataset, which could impact the model’s predictive results, data augmentation was implemented to balance the dataset. For each dataset, we increased the number of images through cropping, flipping, and translation, equalizing the image quantity across datasets to effectively prevent model overfitting caused by data imbalance. In this study, both the baseline models and the DSEception model were trained and tested using datasets that had undergone data preprocessing and augmentation. This approach ensured fair comparison results and effectively enhanced the models’ performance.

### Model construction

#### DSEception model

The Inception architecture was introduced by Szegedy et al. (2014) in 2014, utilizing the concept of “Network In Network” to implement a novel convolution operation known as the “Inception module”. This module enables the model to capture information at various scales while maintaining parameter efficiency, allowing for the extraction of image features across different scales within a single module. Owing to its excellent generalization ability and predictive capability, the Inception model has been widely applied in processing various medical images (Gao et al., 2020; Wang et al., 2019; Tamilarasi and Gopinathan, 2021; Fan et al., 2023). However, the Inception model’s capacity to capture certain detail information in lung X-ray images is somewhat limited, constraining the model’s predictive performance to an extent. Depthwise Separable Convolution (DSC), proposed by Sifre et al. (Sifre and Mallat, 2014) in 2014, has been shown to reduce the model’s computational complexity and the number of parameters while maintaining or even enhancing model performance and extracting richer texture features. The Squeeze-and-Excitation (SE) mechanism, introduced by Hu et al. (Jie et al., 2019) in 2017 as an innovative network architecture aimed at improving the performance of convolutional neural networks, allows for dynamic feature recalibration. This adaptive recalibration process enhances the model’s ability to distinguish the importance of different features, thereby reallocating feature attention regions and effectively improving model prediction accuracy.

**FIGURE 2:**
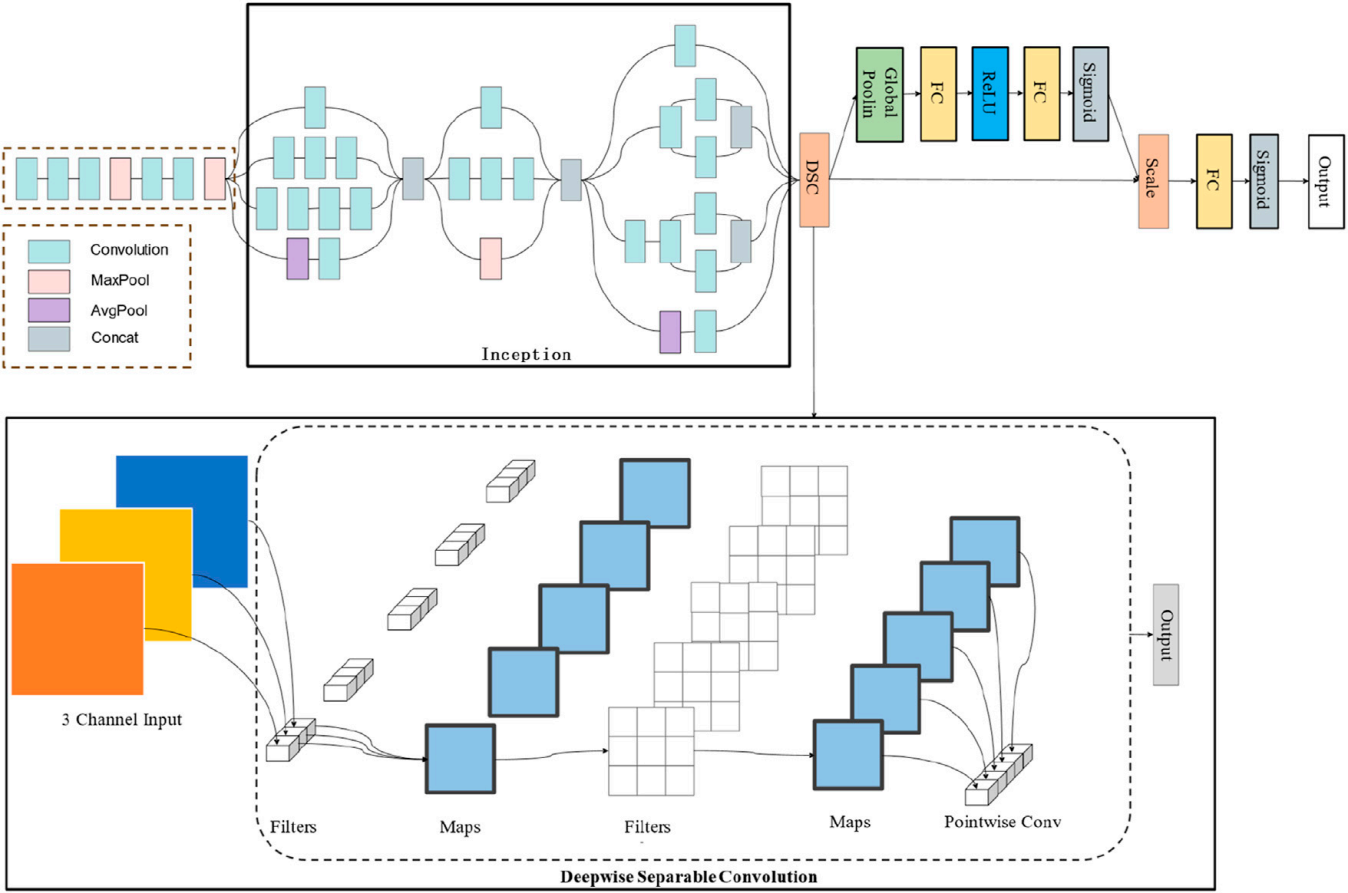
The construction of DSEception model. Initially, the model employs the Inception architecture to perform preliminary feature extraction. Subsequently, it utilizes a Deepwise Separable Convolution structure to extract detailed texture features. Furthermore, the Squeeze-and-Excitation blocks are integrated to adjust the model’s attentional focus without significantly increasing the number of parameters.

The DSEception model proposed in this paper is based on the Inception architecture, incorporating the DSC module to reshape the feature map size while extracting more detailed texture features without significantly increasing the number of parameters. Moreover, the model integrates the Squeeze-and-Excitation mechanism from SENet, a lightweight self-attention mechanism that enhances the model’s representational ability by learning the importance of different channels and readjusting the distribution of feature importance. The construction of DSEception model is shown in Figure 2. These innovations enable DSEception to extract richer features and enhance the detection capability for lung X-ray images, focusing on both broad tissue structures and minute pathological details, significantly improving the model’s performance in diagnosing and classifying lung diseases (pneumonia, tuberculosis, and normal lung conditions).

#### Baseline models

To objectively and comprehensively assess the DSEception model presented in this study, we constructed a set of widely utilized baseline models for comparison, encompassing Inception, VGG, ResNet, and DenseNet. ResNet, proposed by He et al. (2016) in 2015, innovates with the introduction of residual blocks, each comprising one or more convolutional layers, augmented with a “shortcut connection” or “skip connection” between these layers’ in put and output. This design aids in learning the residual mappings between inputs and outputs, thereby alleviating the vanishing gradient issue and facilitating the construction of deeper networks. Due to its capability to capture finer semantic details, ResNet has found extensive application in medical imaging research (Muhammad et al., 2023; Xu et al., 2023).

The VGG network, introduced by the Visual Geometry Group at the University of Oxford in 2014 (Simonyan and Zisserman, 2014), despite its parameter intensity and computational demands, is celebrated for its architectural simplicity and exemplary performance, serving as a critical benchmark in the realm of deep learning applications. Its utility extends beyond medical image recognition to encompass tasks like image segmentation, credited to the formidable expressive power of its feature extraction layers (Johansen et al., 2020; Veni and Manjula, 2023).

DenseNet, unveiled by Huang et al. (2016) in 2016, distinguishes itself by ensuring each layer receives inputs from all preceding layers, thereby fostering dense inter-layer connections. This structure is particularly advantageous for image recognition and classification tasks, demonstrating exceptional efficacy across various medical imaging applications. The juxtaposition with these established baseline models illuminates the DSEception model’s advantages and areas for enhancement, offering profound insights for our investigation’s progression (Huang et al., 2020; Yang et al., 2021).

#### Model evaluation

**FIGURE 3:**
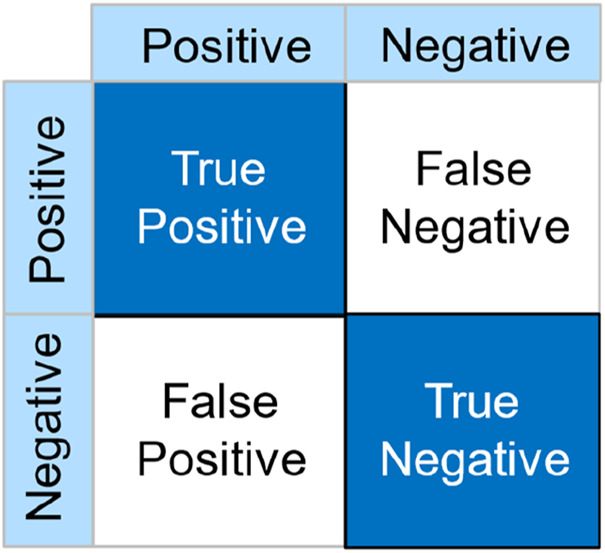
Schematic diagram of how the confusion matrix is calculated.

In this study, we employed several evaluation metrics to assess and compare the performance of our model. These metrics include accuracy (ACC), precision (PRE), recall (REC), and F1-score (F1). Each of these metrics is calculated based on parameters derived from the confusion matrix, which consists of True Positives (TP), True Negatives (TN), False Positives (FP), and False Negatives (FN). The calculation and interpretation of these metrics are essential for understanding the model’s performance in terms of correctly identifying positive cases and avoiding false detections. Figure 3 illustrates the structure of the confusion matrix used in these calculations.

• ACC is the ratio of correctly classified instances to the total number of instances. It represents the model’s overall performance in terms of its ability to correctly classify both positive and negative instances. The formula for calculating accuracy is shown in Equation 1.
• PRE measures the accuracy of positive predictions, indicating the proportion of true positive instances among all instances that the model classified as positive. The formula for calculating precision is provided in Equation 2.
• REC indicates the model’s ability to capture positive instances, reflecting the proportion of actual positive instances that the model correctly identified. The formula for calculating recall is given in Equation 3.
• The F1 Score is the weighted average of precision and recall, providing a balance between these two metrics. It is particularly useful when the class distribution is imbalanced. The formula for calculating the F1 Score is illustrated in Equation 4.

$$ACC=\frac{TP+TN}{TP+FP+TN+FN}$$ (1)

$$PRE=\frac{TP}{TP+FP}$$ (2)

$$REC=\frac{TP}{TP+FN}$$ (3)

$$F1=\frac{2\times PRE\times REC}{PRE+REC}$$ (4)

In addition, we utilized the ROC curve to visualize the model’s performance and computed the Area Under Curve (AUC). The ROC (Receiver Operating Characteristic) curve is an instrumental graphical tool for evaluating the performance of classification models across all possible thresholds. It plots the True Positive Rate (TPR) against the False Positive Rate (FPR). The TPR represents the model’s ability to correctly identify positive samples, whereas the FPR indicates the frequency of incorrectly identifying negative samples as positive. An ideal ROC curve would approach the top left corner of the plot, signifying a low FPR and a high TPR, which is desirable in most classification contexts. The AUC, quantifying the overall performance of a classifier, represents the area under the ROC curve, with values ranging from 0 to 1. An AUC value of one indicates perfect classification, 0.5 suggests no discriminative ability (equivalent to random guessing), and values less than 0.5 imply performance worse than random guessing.

## Results

### Experimental set up

In this study, to ensure a fair and objective evaluation of the models, a uniform fine-tuning and optimization experiment was applied across all models. Specifically, the number of training epochs was set to 30 for all models, under which condition each model achieved full convergence without showing signs of overfitting. For the DSEception Model, the learning rate was adjusted to 0.0001 using the gradient threshold method, and the batch size was set to 32. Under these parameter settings, the model demonstrated optimal predictive performance. Similarly, for other baseline models, equivalent strategies for parameter settings were employed to ensure that each model could perform at its best.

The experimental framework for this investigation was meticulously configured within a computing milieu governed by Windows 11 Professional Edition. Computational operations were conducted utilizing Python 3.7.0. The cornerstone libraries harnessed in this study encompassed Tensorflow-gpu 2.6.0 for the crafting of deep learning architectures, alongside Scikit-learn and Sklearn 0.0.post1 for the implementation of machine learning algorithms and data manipulation, scipy 1.10.0 for advanced scientific computations, and matplotlib for the graphical representation of data and exhibition of results. From a hardware perspective, the experimental activities were executed on an Intel Core i5 12400F CPU, which boasts a base frequency of 2.5 GHz and can achieve a turbo frequency of up to 4.40GHz, incorporating six cores and twelve threads. This CPU was augmented by an NVIDIA GeForce GTX 3060 GPU, which is furnished with a 12 GB memory capacity and a 192-bit memory bus width, thereby ensuring expeditious data handling and model training processes.

### Result of five-fold cross-validation

**FIGURE 4:**
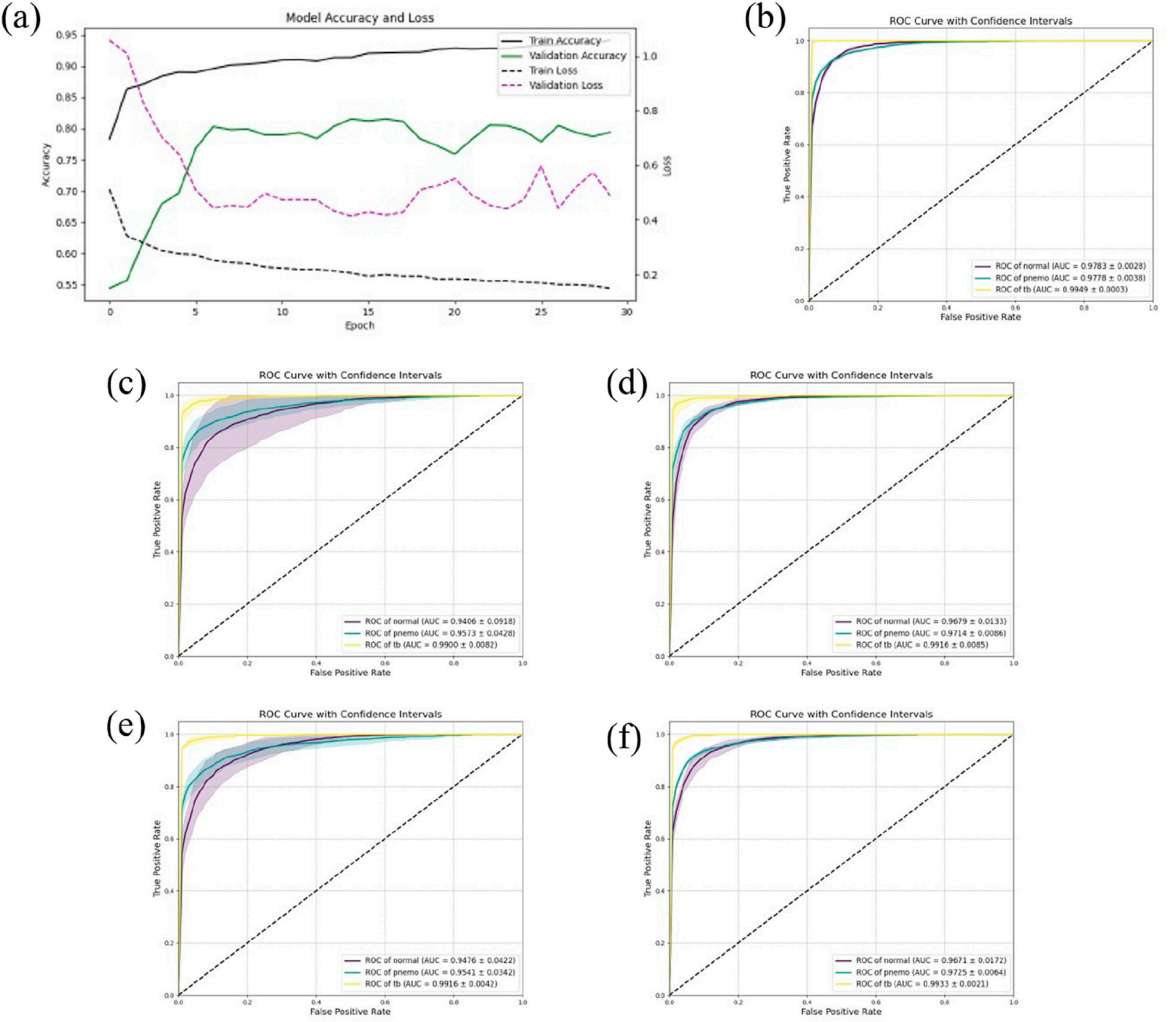
The results of Five-Fold Cross-Validation. **(A)** the training flow of DSEception Model. As can be seen from the figure, as the model is continuously iterated, the training and validation accuracy continue to increase, and the loss continues to decrease until it reaches stability. This shows that the model has converged and there is no overfitting. **(B)** the ROC curve of DSEception Model. **(C)**–**(F)** the ROC curve of DenseNet, Inception, ResNet and VGG. From the above ROC curves, it can be seen that the AUC of the DSEception Model represented by **(B)** in the recognition of pneumonia, tuberculosis and normal is higher than that of the comparison model **(C–F)**.

In this study, to comprehensively assess the predictive accuracy and stability of the models, five-fold cross-validation was conducted on all models. Specifically, for the DSEception model during the training phase, the Adam optimizer was utilized, with the learning rate set to 0.0001 and the number of training epochs fixed at 30. Under these parameter settings, the model was able to fully converge without showing any signs of overfitting, as depicted in Figure 4A.

The results from the five-fold cross-validation (presented in Table 1) demonstrated that the DSEception model exhibited outstanding predictive performance, with average ACC, PRE, REC, and F1 score of 90.98% ± 1.48%, 91.02% ± 2.91%, 93.42% ± 0.83%, and 91.94% ± 2.11%, respectively. Additionally, the AUC for each classification exceeded 97%, with the AUC for tuberculosis reaching 99.49% ± 0.03%, showcasing the model’s exceptional stability (as shown in Figure 4B). In contrast, the ROC curves for the baseline models (illustrated in Figures 4C–F), with the best-performing being Inception, had average ACC, PRE, REC, and F1 scores of 88.27% ± 2.76%, 89.29% ± 2.66%, 90.23% ± 2.72%, and 89.29% ± 2.69%, respectively, all of which were more than 1% lower than those of the DSEception model. Compared to widely used traditional models, the superior predictive capabilities and stability of the proposed DSEception model significantly enhance the trust of medical practitioners and patients in this model.

**TABLE 1:** The results of Five-Fold Cross-Validation.

	Mean ACC (%)	Mean PRE (%)	Mean REC (%)	Mean F1 (%)
DSEception	90.98% ± 1.48	91.02% ± 2.91	93.42% ± 0.83	91.94% ± 2.11
Inception	88.27% ± 2.76	89.29% ± 2.66	90.23% ± 2.72	89.29% ± 2.69
VGG	81.65% ± 11.24	79.95% ± 8.78	86.21% ± 7.21	78.52% ± 12.11
ResNet	83.30% ± 8.63	79.93% ± 7.69	87.88% ± 5.78	80.37% ± 10.10
DenseNet	88.91% ± 0.97	86.67% ± 3.18	90.63% ± 0.35	88.24% ± 2.00

### Results of independent validation

**FIGURE 5:**
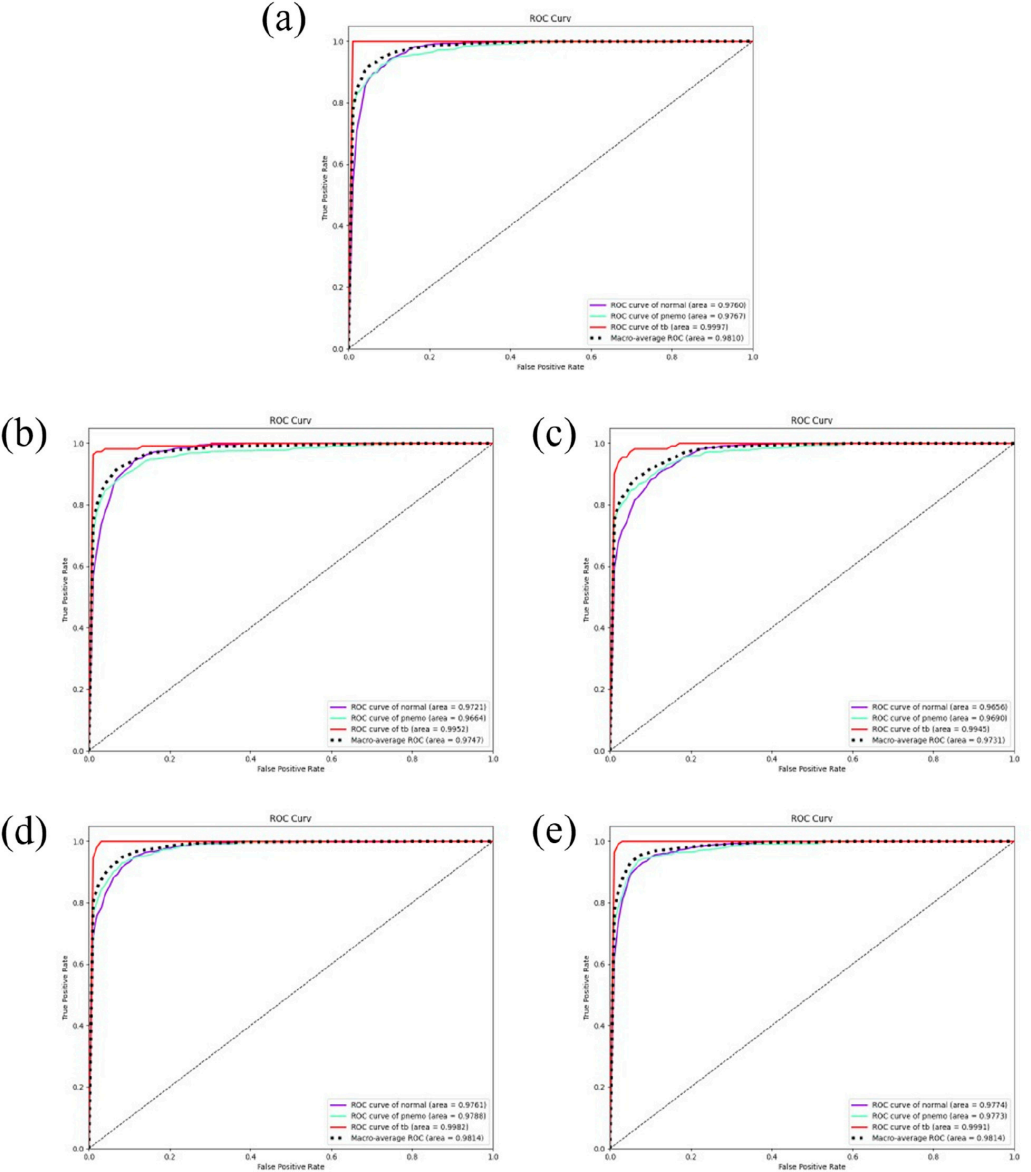
The results of independent validation. **(A)** the ROC curve of DSEception Model. **(B)**–**(E)** the ROC curve of DenseNet, Inception,ResNet and VGG. It can be seen that the average AUC of DSEception Model is similar to or slightly higher than that of other models.

To assess the predictive capability of the DSEception model proposed in this study on unseen datasets, external testing was conducted and compared with baseline models. The results from the external testing underscore the DSEception model’s exceptional ability to maintain high predictive performance when confronted with unseen datasets. The ROC curve of the DSEception model in external testing, as depicted in Figure 5A, achieved an average AUC of 98.10%. Although this is not the highest, it is not significantly different from the other models. Furthermore, the predictive outcomes for all models, as shown in Table 2, indicate that the DSEception model’s ACC, PRE, REC, and F1 score reached 90.48%, 90.07%, 93.49%, and 91.44%, respectively, marking an improvement of over 3% compared to the baseline models.

**TABLE 2:** The results of independent validation.

	Mean ACC (%)	Mean PRE (%)	Mean REC (%)	Mean F1 (%)
DSEception	90.48	90.07	93.49	91.44
Inception	84.65	82.08	88.48	83.60
VGG	85.21	79.41	89.13	80.44
ResNet	86.94	85.75	91.08	87.34
DenseNet	84.31	78.61	88.40	79.66

The ROC curves of the baseline models are illustrated in Figures 5B–E, with the best-performing ResNet model having average ACC, PRE, REC, and F1 scores of 86.94%, 85.75%, 91.08%, and 87.34%, respectively. Compared to ResNet and other baseline models, the DSEception model’s superior capability in handling unseen data is particularly noteworthy, demonstrating its potential to assist clinicians in the efficient, accurate, and rapid diagnosis of various types of pulmonary diseases.

### Visualization and clinical interpretive analysis

**FIGURE 6:**
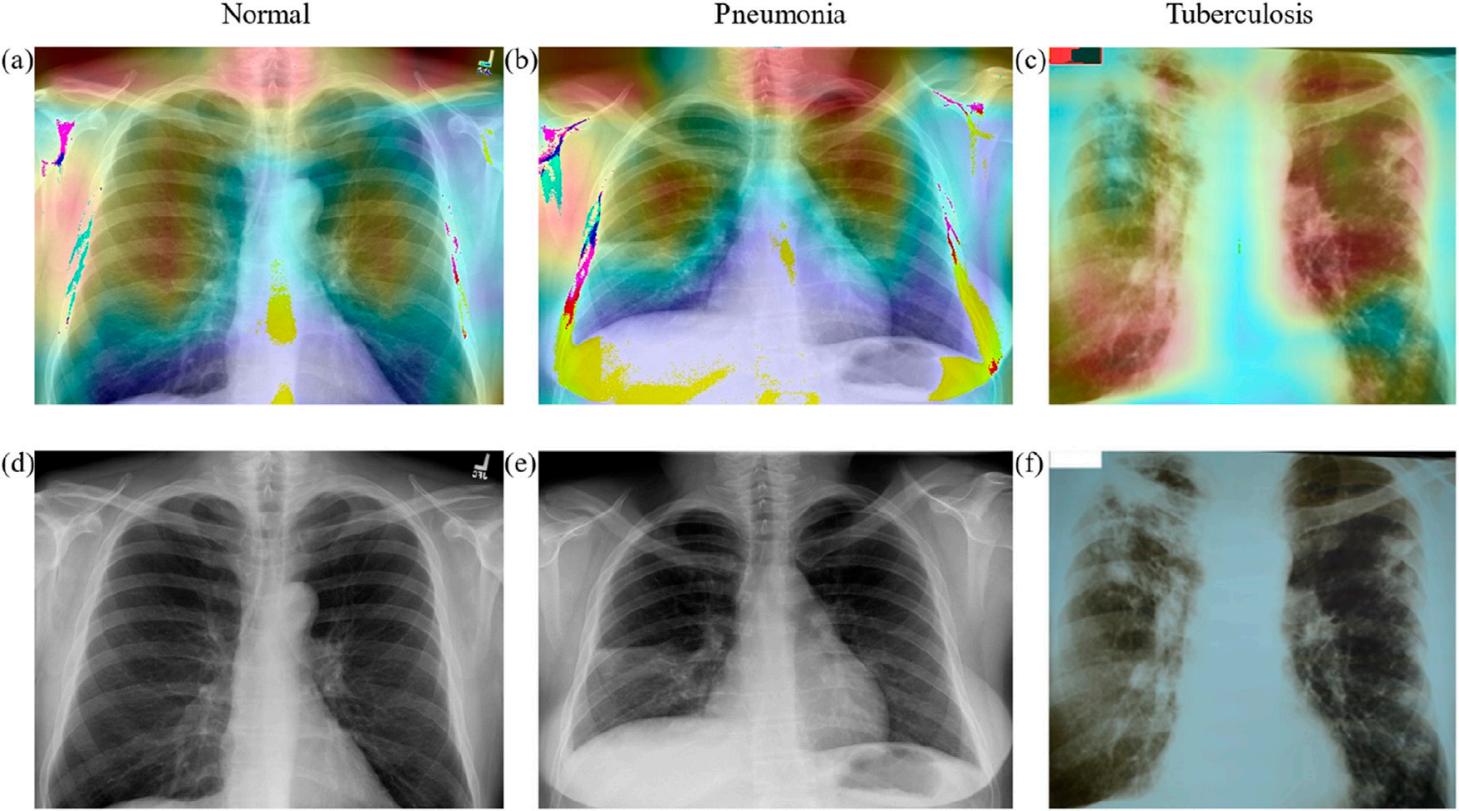
The heatmap of different classes. **(A–C)** the heat map of Normal, Pneumonia, Tuberculosis classes, and **(D–F)** the original X-ray images of Normal, Pneumonia, Tuberculosis classes.

We employed a deep learning model for the analysis of chest X-ray images and generated corresponding heatmaps through visualization techniques to explore the model’s focus areas in identifying different thoracic diseases. As illustrated in Figure 6, the heatmap of a normal chest X-ray image shows uniformly distributed areas of low intensity, indicating no abnormal focal attention by the model. In contrast, for cases of pneumonia and tuberculosis, the heatmaps reveal regions of significantly increased attention, particularly in specific parts of the lungs typically associated with infection and pathological changes. For pneumonia, the heatmap highlights areas of concentrated inflammation, often manifesting as localized increased density in the lung parenchyma. In tuberculosis cases, the focus is on the apical and upper lobe regions, consistent with clinical observations that tuberculosis tends to form lesions in these areas. These results not only confirm the diagnostic consistency of the model but also highlight the potential of deep learning in the interpretive analysis of medical imaging, providing valuable visual aids for clinical decision-making.

## Discussion

This study is dedicated to the precise diagnosis of various pulmonary diseases (Normal, Pneumonia, and Tuberculosis) through the proposed DSEception Model. To evaluate its effectiveness, both five-fold cross-validation and external testing were conducted. In external testing, the DSEception Model demonstrated exceptional performance, with an average accuracy (ACC) and F1 score reaching 90.48% and 91.44%, respectively, marking an improvement of over 3% in accuracy compared to baseline models. During the five-fold cross-validation, the model exhibited superior stability, with an average ACC and F1 score of 90.98% ± 1.48% and 91.94% ± 2.11%, respectively. Hence, due to its high reliability and stability, the DSEception Model is expected to gain widespread trust among medical professionals and patients.

The DSEception model, built upon the InceptionV3 architecture, incorporates depthwise separable convolutions following the Inception modules to enhance parameter efficiency and reduce computational demands. Moreover, the model integrates the SE mechanism from SENet, a lightweight self-attention mechanism that significantly improves the model’s representational ability by precisely learning the importance of different channels without notably increasing the number of parameters and computational load. These innovative approaches enable the Inception modules to extract more complex features more effectively, thus significantly enhancing the model’s performance in diagnosing and classifying pulmonary diseases.

Extensive research has highlighted that traditional manual survey methods in medical diagnosis may be influenced by sampling errors, leading to misdiagnoses (Feng et al., 2014). This issue is prevalent in the context of lung diseases, where the differentiation between various conditions requires precision and accuracy. The mathematical model introduced in this paper addresses this critical gap, offering a more reliable and systematic approach to diagnosis. This model excels in distinguishing between three primary types of lung diseases. Specific characteristics of these diseases might be overlooked in manual evaluations due to complexity in radiological findings. By integrating a complete set of diagnostic criteria and utilizing algorithms, the model increases the accuracy of identifying these diseases, thereby helping medical personnel in making proper healthcare decisions. Furthermore, if the approach proposed in this paper is implemented, hospitals and related medical institutions could significantly reduce medical expenses. Data shows that in regions with relative low economic development, the prevalence of health insurance among residents is low (Durizzo et al., 2022). The diagnostic method proposed in this paper benefits in these resource-limited medical environments: by minimizing unnecessary tests, diagnoses, treatments, and hospital stays, the model optimizes the allocation of medical costs for both hospitals and patients. Mediately, this method improves the overall experience of patients during medical visits. The stress and anxiety associated with long-term and uncertain diagnostic processes can be substantial for patients. A rapid and accurate diagnosis, as streamlined by this model, alleviates these psychological burdens. Therefore, timely and accurate treatment not only saves patients from unnecessary additional medical expenses but also provides stronger support for their mental health, promoting recovery during the illness.

On the other hand, the DSEception model proposed in this study excels in feature extraction and channel importance learning while maintaining a low parameter count and high computational efficiency, making it highly suitable for application in medical electronic products. Medical devices incorporating the DSEception model, such as intelligent imaging diagnostic systems and portable diagnostic tools, can provide rapid and accurate auxiliary diagnoses in clinical settings, particularly in resource-limited or immediate diagnosis scenarios. Specifically, the DSEception model can be embedded in medical imaging analysis instruments like CT or X-ray machines, enabling real-time analysis of imaging data to provide high-precision lesion detection and classification results, thereby assisting physicians in making diagnostic decisions. Furthermore, when combined with smartphones or other portable devices, the DSEception model can be used for telemedicine, facilitating rapid screening and diagnosis for patients in remote areas, thus enhancing the accessibility and efficiency of medical services. Additionally, the lightweight nature of the DSEception model makes it feasible for hardware implementation, allowing it to operate without reliance on high-performance computing equipment. This not only reduces the cost of medical devices but also makes them more accessible, thereby serving a broader range of primary healthcare institutions and home healthcare environments.

The work still has imperfections, which can be specifically discussed in terms of classification limitations, data set scale, and the necessity of clinical translation. Firstly, the current study is only applicable to the specific diagnosis of three types of pulmonary diseases; however, pulmonary diseases have more complexities than this. Therefore, future research could consider including a greater variety of pulmonary disease classifications to enhance the model’s generalization ability and precision in diagnosis. Consequently, the method proposed in this paper would be able to address the complex and varied clinical situations more comprehensively. Secondly, the current research data set used is limited in scale, which may affect the reliability of the model, so future work should include verification on a more extensive data set. Such validation will help ensure that the model can accurately diagnose specific patient conditions in different clinical environments. Ultimately, although this study has made theoretical progress, it has not yet been implemented in the real medical diagnostic process. Future work needs to focus on further developing this theoretical approach into a complete methodology that can be integrated into the current medical system, further developing the potential value of this research.

## Conclusion

In summation, a novel deep learning model for the automated differentiation of pneumonia and tuberculosis using X-ray imaging has been pioneered in this study. The model’s performance in accuracy and reliability, as evidenced through rigorous cross-validation and independent testing, marks a significant advancement in the field of pulmonary disease diagnosis. The work not only simplifies the diagnostic process, reducing the workload for physicians, but also facilitates the advancement of more accurate diagnostic methodologies for prevalent respiratory diseases. Future efforts may focus on expanding the model’s classification spectrum, enhancing the generalizability with multi-center trials, and achieving clinical implementation. This research holds the promise of changing the approach to diagnosing pulmonary diseases globally, emphasizing the transformative potential of integrating advanced deep learning with medical imaging in healthcare.

## Data Availability

Publicly available datasets were analyzed in this study. This data can be found here: https://www.kaggle.com/datasets/tawsifurrahman/tuberculosis-tb-chest-xray-dataset.
